# An active site–tail interaction in the structure of hexahistidine-tagged *Thermoplasma acidophilum* citrate synthase

**DOI:** 10.1107/S2053230X15015939

**Published:** 2015-09-23

**Authors:** Jesse R. Murphy, Stefano Donini, T. Joseph Kappock

**Affiliations:** a Department of Biochemistry, Purdue University, 175 South University Street, West Lafayette, IN 47907-2063, USA

**Keywords:** thermophile, euryarchaeon, conformation change, carbon–carbon bond formation

## Abstract

Citrate synthase (CS) plays a central metabolic role in aerobes and many other organisms. The CS reaction comprises two half-reactions: a Claisen aldol condensation of acetyl-CoA (AcCoA) and oxaloacetate (OAA) that forms citryl-CoA (CitCoA), and CitCoA hydrolysis. Protein conformational changes that ‘close’ the active site play an important role in the assembly of a catalytically competent condensation active site. CS from the thermoacidophile *Thermoplasma acidophilum* (TpCS) possesses an endogenous Trp fluorophore that can be used to monitor the condensation reaction. The 2.2 Å resolution crystal structure of TpCS fused to a C-terminal hexahistidine tag (TpCSH6) reported here is an ‘open’ structure that, when compared with several liganded TpCS structures, helps to define a complete path for active-site closure. One active site in each dimer binds a neighboring His tag, the first nonsubstrate ligand known to occupy both the AcCoA and OAA binding sites. Solution data collectively suggest that this fortuitous interaction is stabilized by the crystalline lattice. As a polar but almost neutral ligand, the active site–tail interaction provides a new starting point for the design of bisubstrate-analog inhibitors of CS.

## Abstract 2

Citrate synthase from the thermophilic euryarchaeon *T. acidophilum* fused to a hexahistidine tag was purified and biochemically characterized. The structure of the unliganded enzyme at 2.2 Å resolution contains tail–active site contacts in half of the active sites.

## 1. Introduction   

Citrate synthase (CS) performs two sequential reactions: a reversible condensation reaction converts acetyl coenzyme A (AcCoA) and oxaloacetate (OAA) into citryl-CoA (CitCoA), and an irreversible thioester hydrolysis then forms citrate and CoA. This pivotal metabolic reaction is performed by members of at least three enzyme superfamilies (Eggerer, 1965 ▸; Gottschalk & Barker, 1966 ▸; Kobylarz *et al.*, 2014 ▸).

The CS dimer is a classic case of induced-fit substrate binding (Srere, 1966 ▸; Bloxham *et al.*, 1980 ▸). OAA binding induces domain closure and the formation of an AcCoA binding site between the large and small domains of each subunit (Remington *et al.*, 1982 ▸; Wiegand & Remington, 1986 ▸; Daidone *et al.*, 2004 ▸). Since many conserved active-site residues participate in both the condensation and hydrolysis reactions, the central CS–CitCoA complex is expected to toggle among multiple configurations (Bayer *et al.*, 1981 ▸). Strong selective pressure ensures a high degree of substrate specificity: CS does not cleave AcCoA but efficiently hydrolyzes CitCoA (Srere, 1972 ▸).

A mechanism proposed for the condensation reaction, based in part on the crystal structures of liganded complexes (Karpusas *et al.*, 1990 ▸; Remington, 1992 ▸), remains broadly consistent with subsequent experimental findings. Computation-based models for the proton transfers involved in carbon–carbon bond formation (Donini *et al.*, 2000 ▸; Mulholland *et al.*, 2000 ▸; Yang & Drueckhammer, 2003 ▸; van der Kamp *et al.*, 2008 ▸, 2010 ▸) have not reached consensus (Aleksandrov *et al.*, 2014 ▸).

CS from the thermophilic, acidophilic euryarchaeon *Thermoplasma acidophilum* (TpCS; Danson *et al.*, 1985 ▸) has several advantages for mechanistic studies, among them an endogenous fluorophore that reports directly on enzyme chemistry (Kurz *et al.*, 2000 ▸, 2005 ▸). TpCS Trp348 fluorescence is strongly quenched by OAA binding nearby (Kurz *et al.*, 2005 ▸). The subsequent addition of either carboxymethyl-CoA (CMCoA) or carboxymethyl-dethiaCoA (CMX), potent CS inhibitors that resemble the deprotonated AcCoA species produced during the condensation reaction, forms a ‘closed’ ternary complex (Bayer *et al.*, 1981 ▸; Karpusas *et al.*, 1990 ▸; Kurz *et al.*, 1992 ▸) but has little effect on TpCS fluorescence (Kurz *et al.*, 2005 ▸). Quenching is, however, alleviated by the subsequent addition of dethiaacetyl-CoA (AcMX), an AcCoA analog that contains a methylene instead of sulfur (Martin *et al.*, 1994 ▸; Kurz *et al.*, 1997 ▸, 2005 ▸). The fluorescence increase is owing to destruction of the Trp348 quencher, the polarized OAA carbonyl, by its conversion to an *sp* ^3^ carbon in the non­hydrolyzable CitCoA analogue dethiacitryl-CoA (CitMX; Kurz *et al.*, 2009 ▸). Reversible, stoichiometric formation of a nonhydrolyzable binary TpCS–CitMX complex was detected by equilibrium fluorescence analysis, pre-steady-state kinetics and a crystal structure (PDB entry 2r9e; C. Lehmann, L. C. Kurz & T. E. Ellenberger, unpublished work). Since the TpCS protein conformation is almost the same in the ternary TpCS–OAA–CMCoA complex (PDB entry 2r26; C. Lehmann, L. C. Kurz & T. E. Ellenberger, unpublished work), the active site arrangement is likely to represent the configuration used for the condensation reaction, not hydrolysis.

This study addresses two barriers to the further study of conformational changes in TpCS. Firstly, an unliganded structure of TpCS has been reported (Russell *et al.*, 1994 ▸) but the coordinates have not been deposited. This hampers the analysis of protein motions accompanying the formation of the TpCS–OAA (PDB entry 2ifc; C. Lehmann, L. C. Kurz & T. E. Ellenberger, unpublished work) and TpCS–OAA–CMCoA complexes. Secondly, TpCS purification involves a dye-linked affinity column (Sutherland *et al.*, 1991 ▸) that may displace (acyl-)CoA ligands (Weitzman & Ridley, 1983 ▸) and irreversibly binds at least one mutant protein (Constantine, 2009 ▸). Here, we report the purification, characterization and crystal structure of TpCS fused to a C-terminal hexahistidine affinity tag (TpCSH6). While the affinity tag has only minor effects on enzyme function in solution, it promotes the formation of a crystal with a His tag bound to an adjacent active site. Liganded and ‘empty’ partner subunits adopt essentially the same ‘open’ conformation.

## 2. Materials and methods   

### 2.1. Macromolecule production   

*Escherichia coli* C41(DE3) cells transformed with either pJK438 or pJK511 (cloning procedures are given in the Supporting Information) were propagated in LB medium supplemented with 50 mg l^−1^ kanamycin. Production cultures (1 l) were grown at 310 K to an optical density at 600 nm of 0.6, at which point isopropyl β-d-1-thiogalactopyranoside (IPTG; Gold Biotechnology, St Louis, Missouri, USA) was added (0.4 m*M* final concentration) to induce protein production. After a further 16 h, the cells were harvested by centrifugation (8000*g* for 15 min at 277 K), resuspended in 8 ml buffer *H* (20 m*M* Tris–HCl pH 8.0, 100 m*M* KCl) per gram of wet cell weight and disrupted by sonication. Streptomycin was added to a final concentration of 1%(*w*/*v*) from a 10% stock and solids were removed by centrifugation (30 000*g* for 10 min at 277 K). Column-chromatography steps were performed at 295 K under gravity flow. An Ni^2+^-loaded iminodiacetic acid Sepharose column (Sigma–Aldrich, St Louis, Missouri, USA; 2.5 × 3.5 cm, 14 ml column volume) was washed with buffer *H* (140 ml) and the cleared cell lysate was applied. The column was washed with buffer *H* containing 20 m*M* imidazole (42 ml) and was developed with buffer *H* containing 250 m*M* imidazole (100 ml). Protein-containing fractions were pooled and concentrated to >5 mg ml^−1^ by ultrafiltration using an Amicon Ultra-15 centrifugal filter unit (EMD Millipore, Billerica, Massachusetts, USA). Solid ammonium sulfate was added to 85% saturation at 277 K over 30 min. After stirring for a further 30 min at 277 K, aliquots were taken and stored as a slurry at 277 K. For crystal production, an aliquot was dissolved in a minimal volume of TE (20 m*M* Tris–HCl pH 8.0, 1 m*M* EDTA) and applied onto a Sephadex 200 gel-filtration column (2.5 × 28 cm; Pharmacia, Uppsala, Sweden) that was developed in TE. Protein-containing fractions were collected, pooled and concentrated to ∼5 mg ml^−1^. Exchanged samples were kept at 277 K and used within 12 h. Proteins were quantitated by the method of Bradford using bovine serum albumin as a standard (Bradford, 1976 ▸). Gel-filtration analysis was used to determine molecular sizes as described by Mullins *et al.* (2013 ▸). Macromolecule-production information is summarized in Table 1 ▸.

Enzyme activities were determined using a continuous assay that detects CoA release by monitoring the cleavage of 5,5′-dithiobis(2-nitrobenzoic acid) (DTNB) at 412 nm (Δ∊ = 14.1 m*M* ^−1^ cm^−1^; Srere *et al.*, 1963 ▸; Srere, 1969 ▸; Riddles *et al.*, 1979 ▸). One unit (U) is defined as the amount of enzyme required to produce 1 µmol of product per minute. Additional information on biochemical characterization is given in the Supporting Information.

### 2.2. Crystallization   

Sparse-matrix crystallization screens performed using the Wizard I and II kits (Emerald Bio) yielded two hits after 27 d: condition I-1 [20% PEG 8000, 0.1 *M* 2-(cyclohexylamino)­ethanesulfonate (CHES) pH 9.5] and condition I-26 (Table 2 ▸), each supplemented with 1 m*M* OAA. Crystals with a similar appearance were obtained in the same buffer after 10 d in drops (2–4 µl prior to mixing with an equal volume of reservoir solution) containing 8–10% PEG 4000 and 5 mg ml^−1^ TpCSH6 with or without OAA. Single crystals were loaded into nylon loops (Teng, 1990 ▸), transferred to reservoir solution supplemented with 15%(*w*/*v*) sorbitol for 10 min, rapidly immersed in liquid nitrogen and maintained at or below ∼100 K until data collection was complete.

### 2.3. Data collection and processing   

All screened crystals diffracted X-rays weakly and with high mosaicity. The best diffraction patterns were obtained from a TpCSH6 crystal that adhered to the side of the mounting loop. The unusual sample geometry hampered the collection of a complete X-ray diffraction data set, which was recorded in three passes using different regions of the crystal. Diffraction data from one-pass, two-pass or three-pass sets were processed using the *HKL*-2000 suite (Otwinowski & Minor, 1997 ▸). Single-pass data sets had similar statistics (not shown), including individual *R*_sym_ values that were comparable to the *R*_merge_ value for the full (three-pass) data set (Table 3 ▸).

### 2.4. Structure solution and refinement   

*PHENIX* and *Coot* were used for structure solution and refinement (Emsley *et al.*, 2010 ▸; Adams *et al.*, 2010 ▸). Molecular replacement was performed with *Phaser* (McCoy *et al.*, 2007 ▸) using subunit *A* from PDB entry 2ifc, with all buffer components and side-chain atoms beyond C^β^ removed, as the search model. The sole solution contained four subunits in the asymmetric unit (residues 4–383; the TpCS numbering excludes Met0). One round of *phenix.autobuild* (with default settings) was performed to repair side chains. Iterative cycles of model improvement and refinement, using noncrystallographic symmetry (NCS) restraints and a hybrid TLS–isotropic ADP model, were performed until *R*_free_ appeared to converge. The C-terminal appendage (residues Val386–His399) was added using *Coot*. NCS restraints were dropped in the final refinement cycles. *MolProbity* was used to check the protein geometry (Chen *et al.*, 2010 ▸).

Pairwise alignments and root-mean-square deviation (r.m.s.d.) computations were performed using one cycle of the *align* algorithm implemented in *PyMOL* (v.1.7.4.2; DeLano, 2002 ▸). Domain motions were computed using the *DynDom* server (v.1.5; default settings; Hayward & Berendsen, 1998 ▸; Hayward & Lee, 2002 ▸). Protein images were based on *PyMOL* or *LIGPLOT* (Wallace *et al.*, 1995 ▸) output.

## 3. Results and discussion   

### 3.1. Comparison of TpCSH6 and TpCS   

TpCSH6 was overexpressed in *E. coli*, purified and found to be a dimer (Supplementary Fig. S1) with a specific activity of 52 U mg^−1^ at 328 K and ≤22 U mg^−1^ at 298 K (8 s^−1^, assuming one active site per subunit). Substrate-saturation analyses (Supplementary Fig. S2) yielded Michaelis constant (*K*_M_) values of 3.0 µ*M* for AcCoA and 5.2 µ*M* for OAA. AcMX was a competitive inhibitor *versus* AcCoA (*K*_i_ = 20 µ*M*; Supplementary Fig. S3). These results, and the relatively high batch-to-batch variability in specific activity, are comparable to those for TpCS (Sutherland *et al.*, 1991 ▸; Kurz *et al.*, 2000 ▸).

Purified TpCSH6-H222Q, a mutant located in the AcCoA binding site, had a specific activity of ≤0.01 U mg^−1^ at 298 K or ≤0.05% of that of the wild type. Since the mutant enzyme was isolated from a *gltA* ^+^ *E. coli* strain, the observed activity could be associated with a contaminating host enzyme. However, the ‘activity’ did not increase as AcCoA was varied from 30 to 400 µ*M* (at 0.4 m*M* OAA; data not shown), which suggests that host CS is not present. We conclude that TpCSH6-H222Q is nearly or completely devoid of enzymatic activity, and that the protein-isolation method can be used to study even very low-activity mutants produced in common protein-production strains.

Fluorescence titrations were used to measure OAA dissociation constants (*K*_d_) of 0.76 and 19 µ*M* for TpCSH6 and TpCSH6-H222Q, respectively (Supplementary Fig. S4). Saturating OAA quenched the fluorescence of both forms to about half of the initial level, which differs from a report that TpCS-H222Q fluorescence is quenched to a relatively greater extent (Kurz *et al.*, 2009 ▸). As observed for TpCS, the addition of AcMX to the TpCSH6–OAA complex results in a 124% increase in fluorescence (*K*_d_ = 1.5 µ*M*) owing to the formation of CitMX (Supplementary Fig. S5). The *K*_d_ values determined for untagged TpCS(-H222Q) at 293 K were comparable: 1.3, 33 and 1.47 µ*M*, respectively (Kurz *et al.*, 2005 ▸, 2009 ▸).

### 3.2. Crystal structure of TpCSH6   

Initial attempts to crystallize TpCSH6 focused on HEPES/sodium acetate (pH 7.5–8.5) conditions, identified by robotic screening, that yielded crystals of TpCS bound to one or more ligands (Christopher Lehmann, unpublished observations). No suitable TpCSH6 crystals were obtained in attempts to manually reproduce these conditions. A sparse-matrix screen identified high-pH conditions that yielded large crystals (Table 2 ▸). The inclusion of OAA in the crystallization solution (Table 2 ▸) had no obvious effect on crystal morphology. A cryocooled crystal (0.1 × 0.1 × 1.0 mm) grown in the presence of OAA was selected for X-ray data collection. Several attempts to crystallize TpCSH6-H222Q under similar conditions yielded only microcrystals.

The three-pass data set was selected for further analysis because the completeness and electron-density maps (Supplementary Fig. S6) were superior to those computed using the one-pass or two-pass data sets (not shown). Molecular replacement and refinement proceeded smoothly, furnishing a final model with acceptable statistics (Table 4 ▸).

Like TpCS (Russell *et al.*, 1994 ▸), TpCSH6 crystallized in space group *P*2_1_ with two dimers in the asymmetric unit, albeit with rather different unit-cell parameters (Table 3 ▸). TpCSH6 had almost the same monomer topology as unliganded TpCS (Supplementary Fig. S7). Pairwise alignments of TpCS (coordinates provided by Linda C. Kurz) and TpCSH6 sub­units gave r.m.s.d. values of 0.40–0.59 Å (C^α^ only) and 1.15–1.25 Å (all protein atoms). Pairwise alignments of the TpCSH6 subunits (residues 5–383) gave r.m.s.d. values of 0.26–0.42 Å (C^α^ only) and 0.68–0.91 Å (all protein atoms). TpCSH6 subunit *C* and the *CD* dimer were selected to represent the open conformation in all subsequent comparisons.

As expected for a relatively long exposure to synchrotron X-rays, alterations consistent with mild X-ray damage were observed, including Asp/Glu decarboxylation and loss of methanethiol in surface-accessible residues (Garman, 2010 ▸) such as Asp301*B* and Met241*C*. Two well ordered bicarbonate ligands were included in the final model. This was justified by the high pH of the mother liquor and the presence of OAA, which can spontaneously decarboxylate. These anions ‘cap’ the N-terminal (positive) end of the long αQ helix in subunits *B* and *C*. The side chain of the single Ramachandran outlier, Lys237*C*, makes a well defined salt bridge with the side chain of Glu158*D* from a neighboring asymmetric unit.

**Figure 1:**
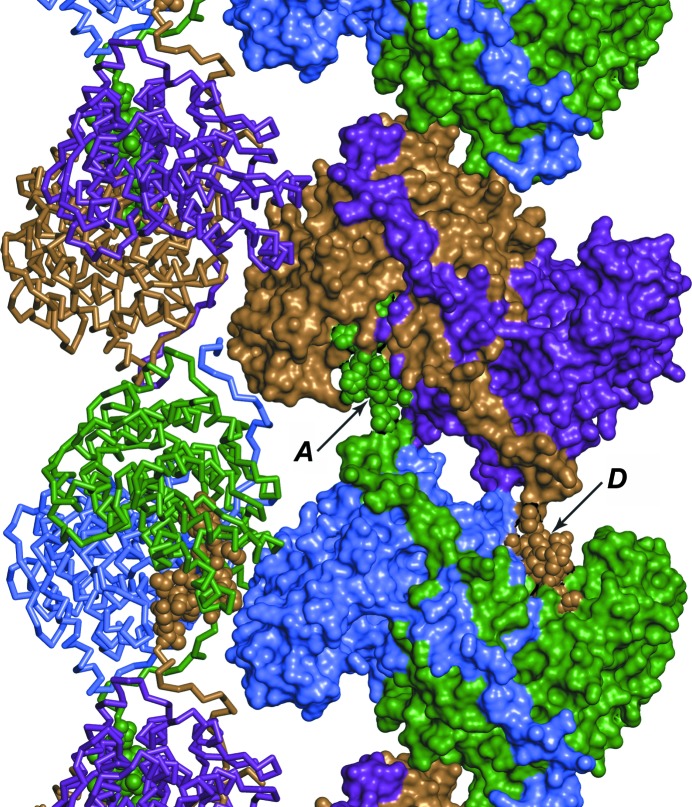
Crystal-packing diagram for TpCSH6. Subunits *A*–*D* in the monoclinic asymmetric unit are colored green, blue, purple and gold, respectively. Residues in the C-terminal appendage, shown as spheres in subunits *A* and *D* (indicated by arrows), occupy the active sites of subunits *D* and *A*, respectively. Left, ribbon rendering shows that the C-termini bind deep within the *AB* and *CD* dimers. In this orientation, the twofold screw axis (*b* axis) runs along the Cartesian *x* axis.

Positive difference electron density was observed early in refinement at two locations: a helix near the *CD* dimer interface and the nearby subunit *D* active site. These features were linked to form the C-terminus of subunit *A*. Dimers *AB* and *CD* are bridged at the center of the asymmetric unit by two C-terminal appendages extending from subunits *A* and *D* (Fig. 1 ▸). The C-terminal native sequence of subunit *A* wraps halfway around the large domain of the *B* subunit. The C-terminal tag sequence continues past helix αT towards the *CD* interface, making a perpendicular turn to form the extra helix (αU). A second sharp turn near residue His222*D* directs the tag sequence into the active site of subunit *D*, ending with a bidentate salt bridge formed between the C-terminal carboxylate of the tag residue His399*A* and the Arg271*D* guanidinium. A similar interaction is formed between the C-terminus of subunit *D* and the active site of subunit *A*. NCS comparisons indicate the backbones diverge after Arg383, with helix αU displaced along its axis about 2 Å farther from the *CD* dimer than the *AB* dimer. No electron density consistent with the C-terminal appendage of subunits *B* or *C* was observed. Small affinity-tag appendages occasionally form crystal-packing contacts (Carson *et al.*, 2007 ▸). The TpCSH6 His_6_ tag is one of a small number of ordered affinity tags that bind to an enzyme active site (Taylor *et al.*, 2005 ▸; McDonald *et al.*, 2007 ▸; Singh *et al.*, 2011 ▸; Wojtkowiak *et al.*, 2012 ▸).

**Figure 2:**
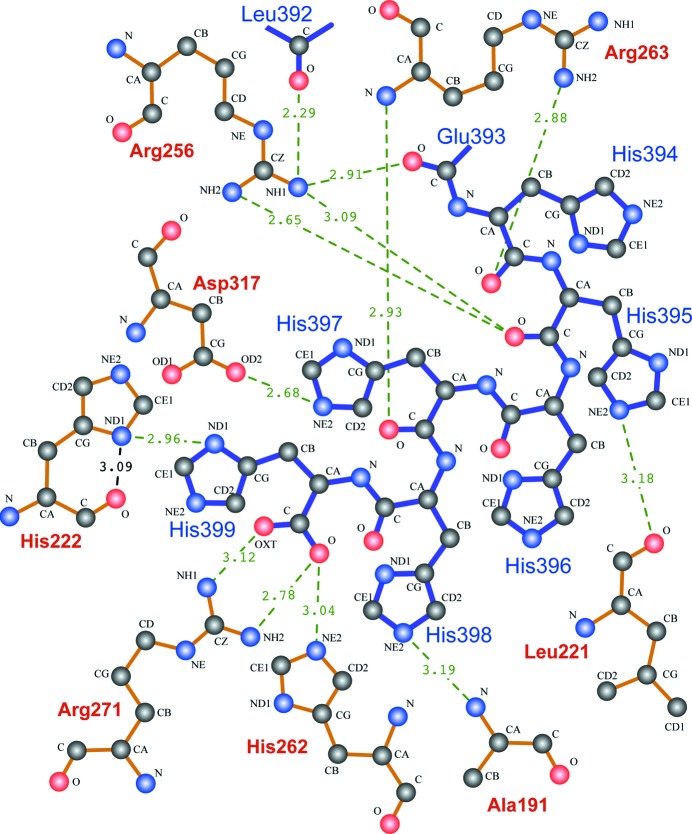
Active site of TpCSH6 subunit *A* bound to the C-terminus of subunit *D* (blue bonds). All illustrated residues also contact OAA (His262 and Arg271) or AcCoA (the remainder). His222 and Asp317 are responsible for deprotonating the AcCoA acetyl group, stabilizing the resulting enolate/carbanion and promoting the condensation reaction that produces CitCoA. An intra-residue hydrogen bond that stabilizes the neutral π tautomer of His222 is shown in black. A similar analysis of the TpCS active site bound to the CitCoA analogue CitMX (Supplementary Fig. S8) shows that many of the same residues are involved in substrate contacts. All polar contacts presumed to result in hydrogen-bonding interactions are shown, with distances in Å.

The C-terminal appendage contacts active-site residues involved in binding both OAA and AcCoA (Fig. 2 ▸). This was unexpected, as the hexahistidine sequence has no resemblance to either substrate. The C-terminal residue His399 makes a side-chain contact with His222, a residue that provides a hydrogen bond (involving N^δ1^) that polarizes the AcCoA enolate, and the terminal carboxylate contacts both His262 and Arg271, which bind carbonyl and α-carboxylate O atoms in OAA. Since the His399–His222 contact involves two N^δ1^ atoms, one residue adopts a π tautomer (N^δ1^–H), which is uncommon at high pH (Sudmeier *et al.*, 2003 ▸; Li & Hong, 2011 ▸) but is employed by some active-site residues (Day *et al.*, 2003 ▸). The local hydrogen-bonding context and the proposed role for the neutral π tautomer in the condensation reaction (summarized in van der Kamp *et al.*, 2007 ▸) suggest that His222 adopts the π tautomer even in the absence of acetyl-CoA. Similarly, flipping the His398 imidazole would worsen the hydrogen-bonding context and introduce clashes, which suggests it may also adopt a π tautomer stabilized by Ala191 NH (Fig. 2 ▸). His397 N^∊2^ forms a hydrogen bond to one carboxylate O atom in the critical active-site base Asp317, which is not directly contacted by either substrate (Supplementary Fig. S8). Similar contacts are observed between Asp317 equivalents and strong inhibitors such as CMCoA and CMX (Usher *et al.*, 1994 ▸; Francois *et al.*, 2006 ▸).

Bisubstrate analogues can be potent and selective enzyme inhibitors (Collins & Stark, 1971 ▸; Radzicka & Wolfenden, 1995 ▸; Schramm, 2013 ▸). Clinically useful examples include finasteride, which is converted to a tight-binding bisubstrate analogue by its target enzyme (Bull *et al.*, 1996 ▸). The stoichiometric ligand CitMX is a bisubstrate analogue inhibitor of CS that (like finasteride) is formed by its target enzyme (Kurz *et al.*, 2009 ▸). Reversible formation of CitMX by TpCS might therefore contribute to inhibition by AcMX (Martin *et al.*, 1994 ▸), although its potency could be sapped by harnessing ‘binding energy’ to drive the condensation reaction (Page & Jencks, 1971 ▸). CS is a highly specific enzyme that possesses a highly basic active site that binds two polyanionic substrates. All known potent CS inhibitors are unpromising starting points for drug design: they closely resemble the substrates and would encounter substantial barriers to cellular uptake. In contrast, imidazoles should be mostly uncharged at the basic pH, which may allow crystal growth by alleviating electrostatic repulsion between the active site and the His_6_ tail. This near-neutral peptide ligand could represent a useful alternative starting point for the identification of CS inhibitors.

To test the hypothesis that the CS active site has detectable affinity for a poly-His ligand, TpCSH6 was incubated with a dansyl-His_3_ synthetic peptide (Genscript, Piscataway, New Jersey, USA). Fluorescence changes consistent with Förster resonance energy transfer between the tripeptide and TpCSH6, presumably the nearby Trp115, were not observed (data not shown).

Gel-filtration studies indicated that unliganded TpCS, TpCSH6 and TpCSH6-H222Q were each exclusively dimeric in solution (Supplementary Fig. S1). Deletion of a hydrogen bond between the active-site residue His222 and the appendage residue His399 appears to thwart TpCSH6-H222Q crystal growth. This sensitivity suggests a weak peptide–protein interaction that is stabilized by cooperative crystal-packing interactions.

### 3.3. TpCS conformational changes associated with ligand binding   

Substrate exclusion by the C-terminal appendage could explain why the crystal morphology was unaffected by the addition of saturating OAA but only half of the active sites are occupied by peptide in the crystal (ordered solvent is observed in the other two active sites). The striking finding that all four subunits adopt open conformations suggests that the TpCS dimer cannot close just one active site. Previous CS crystal structures have a high degree of NCS and contain the same ligand(s) in both active sites. Since to our knowledge there is no evidence for cooperative binding of OAA by any CS, it is possible that the open conformation is trapped within the crystalline lattice.

The preferred rotamer at Trp348, or the residue occupying this position, has been reported to change upon ligand binding and concomitant enzyme closure (Kurz *et al.*, 2005 ▸). The TpCS structure possesses *m*95 (Trp348*A*) and *p*90 (Trp348*B*) rotamers (Russell *et al.*, 1994 ▸), each modeled with a C^α^—C^β^—C^γ^ bond-angle outlier (σ > 5.5). In TpCSH6 and the three deposited TpCS structures, however, only *m*0 rotamers were observed in unambiguous electron density in all subunits (Supplementary Fig. S6). It is possible that binding of the C-terminal appendage to the TpCSH6 active site, which does not cause domain closure, triggers a Trp348 ring flip. A simpler model, however, is that the Trp348 indole does not ring flip, consistent with the fluorescence properties, which indicate a rigidly immobilizing protein environment (Kurz *et al.*, 2005 ▸).

**Figure 3:**
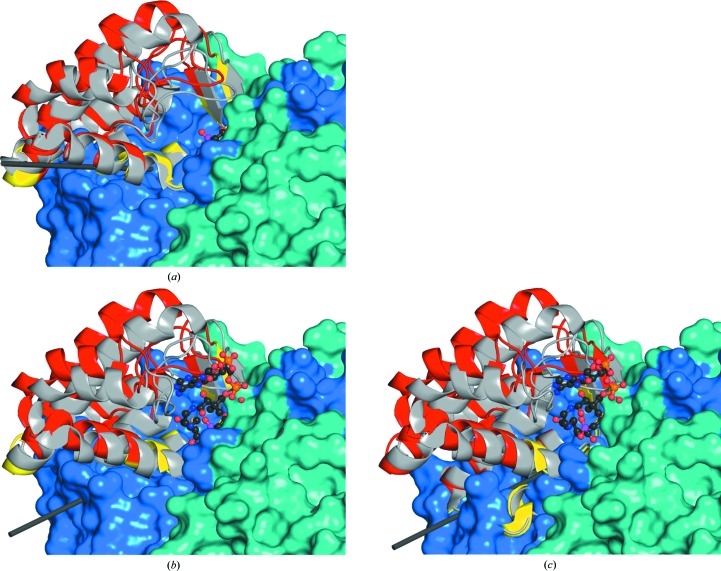
Domain motions in TpCS(H6). Each panel shows the open TpCSH6 structure superimposed on a closed TpCS structure in pairwise *DynDom* analyses. The static portion, mainly the large domains that form the majority of the dimer interface, is shown as a surface rendering. The mobile domain extends from the surface and is shown in cartoon rendering, with the TpCSH6 mobile region colored red and the closed structure colored grey. Hinging residues in each TpCSH6 domain are colored yellow. Residues involved in each pairwise comparison are shown in Supplementary Fig. S7. The rotation axis is shown as a black bar; closure results in clockwise rotations about this axis. (*a*) Comparison with the TpCS–OAA complex (PDB entry 2ifc subunit *A*) shows 52% closure and a 8.9° rotation (translation of 0.1 Å). Mobile domain: 99 residues, r.m.s.d. 0.41 Å. Static domain: 277 residues, r.m.s.d. 0.52 Å. (*b*) Comparison with the TpCS–OAA–CMCoA complex (PDB entry 2r26 subunit *A*) shows 86% closure and a 10.6° rotation (translation of 0.4 Å). Mobile domain: 115 residues, r.m.s.d. 0.68 Å. Static domain: 258 residues, r.m.s.d. 0.69 Å. CMCoA is a tight-binding analogue of the deprotonated AcCoA formed during the condensation reaction. (*c*) Comparison with the TpCS–CitMX complex (PDB entry 2r9e subunit *B*) shows 82% closure and a 10.3° rotation (translation 0.4 Å). Mobile domain: 123 residues, r.m.s.d. 0.83 Å. Static domain: 252 residues, r.m.s.d. 0.7 Å. CitMX is formed from AcMX and OAA, presumably within the crystal.

Using the new TpCSH6 structure as a reference, we examined the series of protein conformational changes associated with ligand binding, domain closure and the condensation reaction (Supplementary Fig. S7). As anticipated, the binding of OAA is associated with a conspicuous closure of the active site: a rotation of the small domain relative to the large domains that form the major dimer interface (Fig. 3 ▸ *a*). Each of the domains is relatively monolithic, with pairwise r.m.s.d. values of <1 Å, and similar sets of hinge residues are involved. This motion is similar to the larger (19.4°) rotation observed in the canonical vertebrate CS open/closed pair (PDB entries 1cts/1csh; Remington *et al.*, 1982 ▸; Usher *et al.*, 1994 ▸; Hayward & Berendsen, 1998 ▸). Domain closure increases when a CoA analogue is also present (Figs. 3 ▸ *b* and 3 ▸ *c*). As noted above, the TpCS–OAA–CMCoA and TpCS–CitMX complexes adopt similar protein structures, suggesting that they both represent the configuration associated with the condensation reaction.

## 4. Conclusions   

TpCSH6 is an active enzyme useful for solution studies of the CS mechanism. It unexpectedly produced a new crystal form that appears to prevent ligand cocrystallization. The serendipitous peptide ligand, with a total charge of near zero, binds both parts of the CS active site. Using this complex as an inspiration, it may be possible to identify an alternative drug scaffold to target this key step in primary metabolism.

## Supplementary Material

PDB reference: citrate synthase, 4ybo

Supporting Information.. DOI: 10.1107/S2053230X15015939/ub5080sup1.pdf

## Figures and Tables

**Table 1:** Macromolecule-production information

Source organism	*T. acidophilum*
DNA source	Plasmid pJK438
Forward primer†	ACAGGAGTACATATGCCAGAAACTGAAGAA
Reverse primer†	TTGAGAAAACTCGAGTCACTTTCTTTCAGC
Expression vector	pET-24a
Expression host	*E. coli* C41(DE3)
Complete amino-acid sequence of the construct produced‡	PETEEISKGLEDVNIKWTRLTTIDGNKGILRYGGYSVEDIIASGAQDEEIQYLFLYGNLPTEQELRKYKETVQKGYKIPDFVINAIRQLPRESDAVAMQMAAVAAMAASETKFKWNKDTDRDVAAEMIGRMSAITVNVYRHIMNMPAELPKPSDSYAESFLNAAFGRKATKEEIDAMNTALILYTDHEVPASTTAGLVAVSTLSDMYSGITAALAALKGPLHGGAAEAAIAQFDEIKDPAMVEKWFNDNIINGKKRLMGFGHRVYKTYDPRAKIFKGIAEKLSSKKPEVHKVYEIATKLEDFGIKAFGSKGIYPNTDYFSGIVYMSIGFPLRNNIYTALFALSRVTGWQAHFIEYVEEQQRLIRPRAVYVGPAERKYVPIAERKVDKLAAALEHHHHHH
UniProt identifier	P21553

† The restriction sites in ODNs 1306 (forward, NdeI) and 1309 (reverse, XhoI) are underlined. Stop codon 385 is replaced by the vector-encoded tag sequence.

‡ The recombinant protein lacks an N-terminal Met. The 15-residue His_6_ tag appended to the C-terminus of the native sequence is underlined.

**Table 2:** Crystallization

Method	Hanging-drop vapor diffusion
Plate type	VDX 24-well tray, Hampton Research
Temperature (K)	294
Protein concentration (mgml^1^)	5
Buffer composition of protein solution	20m*M* TrisHCl pH 8.0, 1m*M* EDTA, 1m*M* oxaloacetate
Composition of reservoir solution	0.1*M* CHES pH 9.5, 12%(*w*/*v*) PEG 4000, 1m*M* oxaloacetate
Volume and ratio of drop	2l (1:1 ratio)
Volume of reservoir (ml)	0.5

**Table 3:** Data collection and processing Values in parentheses are for the outer shell.

Diffraction source	21-ID-G, APS
Wavelength ()	0.97856
Temperature (K)	100
Detector	MAR Mosaic 300mm CCD
Crystal-to-detector distance (mm)	200, 247.8 and 250
Rotation range per image ()	0.5, 1.0 and 1.0
Total rotation range ()	93, 122 and 360
Exposure time per image (s)	3.0, 2.8 and 3.5
Space group	*P*2_1_
*a*, *b*, *c* ()	56.505, 113.406, 120.066
, , ()	90, 95.08, 90
Resolution range ()	502.18 (2.242.18)
Total No. of reflections	983117
No. of unique reflections	76084
Completeness (%)	97.2 (97.0)
Multiplicity	12.9 (13.5)
*I*/(*I*)	11.8 (2.1)
*R*_meas_	0.249 (1.400)
*R*_p.i.m._	0.085 (0.422)
CC_1/2_	0.993 (0.762)
Overall *B* factor from Wilson plot (^2^)	24.9

**Table 4:** Structure refinement Values in parentheses are for the outer shell.

Resolution range ()	29.152.18 (2.242.18)
Completeness (%)	89.8 (76.0)
Cutoff	0
No. of reflections, working set	70618 (4415)
No. of reflections, test set	1867 (126)
Final *R*_cryst_	0.1642 (0.2385)
Final *R*_free_	0.2118 (0.2965)
No. of non-H atoms	
Protein (all atoms)	12251
Protein (His_6_-tag atoms)	277
Ligand	8
Water	868
Total	13127
R.m.s. deviations	
Bonds ()	0.012
Angles ()	1.139
Average *B* factors (^2^)	
Protein (all atoms)	33.8
Protein (His_6_-tag atoms)	63.6
Ligand	45.5
Water	36.7
Ramachandran plot	
Favoured regions (%)	97.93
Additionally allowed (%)	2.01
Outliers (%)	0.06
*MolProbity* clashscore†	4.86
*MolProbity* overall score	1.08

† Clashscore is the number of interatomic overlaps (0.4) per 1000 atoms (Chen *et al.*, 2010 ▸).
