# Supplementary material for: An active site–tail interaction in the structure of hexahistidine-tagged Thermoplasma acidophilum citrate synthase
Source: Acta Crystallogr F Struct Biol Commun. 2015 Sep 23;71(Pt 10):1292–9. doi: 10.1107/S2053230X15015939 (PMC4601594; doi:10.1107/S2053230X15015939)
Supplement: Supplementary file 1 [file f-71-01292-sup1.pdf]

STRUCTURAL BIOLOGY
COMMUNICATIONS

Volume 71 (2015)

Supporting information for article:

An active site-tail interaction in the crystal structure of hexahistidine-tagged *Thermoplasma acidophilum* citrate synthase

Jesse R. Murphy, Stefano Donini and T. Joseph Kappock

Additional experimental methods

DNA manipulations Oligodeoxynucleotides (ODNs) were from IDT (Coralville, IA). DNA-modifying enzymes were from New England Biolabs (Beverly, MA). A Vent DNA polymerase PCR product, obtained with ODN primers 1306 and 1307 (Table 1) and plasmid pRec7-ArCS as template, added flanking *Nde*I and *Xho*I sites to the *gltA* open reading frame and deleted the native stop codon. Ligation into the corresponding sites of plasmid pET24a furnished TpCSH6 expression plasmid pJK438, which adds a 15-residue peptide VDKLA AALEH HHHHH (residues 385-399) to the native C-terminus of TpCS. A QuikChange mutagenesis kit (Agilent, La Jolla, CA), pJK438, ODN 2066 (5′-GCTGCAaGGCGGTGCAGCTGAGG), and ODN 2067 (5′-ACCGCCtTGCAGCGGACCCTTG) were used to construct the TpCSH6-H222Q expression plasmid pJK511. Bidirectional Sanger sequencing of the coding region was used to confirm that both plasmids contained the expected DNA sequences.

Enzyme kinetics and inhibition analysis Enzyme activities at 298 K were recorded on a Cary 100 spectrophotometer (Varian) equipped with a Peltier temperature controller. Nonlinear least-squares fitting was performed using using *gnuplot* (version 4.4).

Enzyme initial velocities were determined in a final volume of 0.7 mL that contained 50 mM 4(2-hydroxyethyl)-1-piperazinepropanesulfonic acid (EPPS), pH 8.0, 0.1 mM EDTA, 0.3 mM DTNB, either 15 μM or 0.4 mM OAA, variable amounts of the other substrate, and either TpCSH6 (0.6 μg) or TpCSH6-H222Q (20 μg). Kinetic constants were obtained by fitting initial velocity data to the Michaelis-Menten equation.

AcMX, synthesized by Hong Jiang as described (Francois *et al.*, 2006), was used to prepare the TpCS $\cdot$ CitMX complex (PDB entry 2r9e) and assessed as a TpCSH6 inhibitor. Initial velocities were measured in a final volume of 0.7 mL that contained 50 mM potassium phosphate, pH 8.0, 100 mM KCl, 0.4 mM OAA, 0.3 mM DTNB, varied AcCoA, and several fixed concentrations of AcMX. Reactions were initiated by the addition of TpCSH6 (0.6 mg). Lineweaver-Burk plots showed a competitive inhibition pattern. All initial velocity data were

globally fit to a Michaelis-Menten equation in which $K_{\mathrm{M,app}} = K_{\mathrm{M}}(1 + [\mathrm{AcMX}]/K_i)$.

Ligand affinity measurements Fluorescence emission spectra (excitation 295 nm) were recorded using a Fluoromax-3 (Horiba) spectrofluorimeter at 298 K. Emission spectra were corrected for dilution and, using a control titration of *N*-acetyl-L-tryptophanamide, the inner filter effect due to OAA. Titration data monitoring changes in the fluorescence emission intensity at 315 nm (F) were plotted by normalizing F by the emission intensity measured in the absence of titrant (F_o).

Protein-ligand affinities were determined by fits to Eqn. S1 (for $K_{\mathrm{d}}/[\mathrm{E}] > 5$, where [E] is the subunit concentration of the enzyme) or Eqn. S2, which accounts for changes in [L] (either [OAA] or [AcMX]) due to ligand depletion.

$$F = F_o + \frac{\Delta F[\mathrm{L}]}{K_{\mathrm{d}} + [\mathrm{L}]} \tag{S1}$$

$$F = F_o + \frac{\Delta F}{2[\mathrm{E}]}\left([\mathrm{L}] + K_{\mathrm{d}} + [\mathrm{E}] + \sqrt{([\mathrm{L}] + K_{\mathrm{d}} + [\mathrm{E}])^2 + 4[\mathrm{L}][\mathrm{E}]}\right) \tag{S2}$$

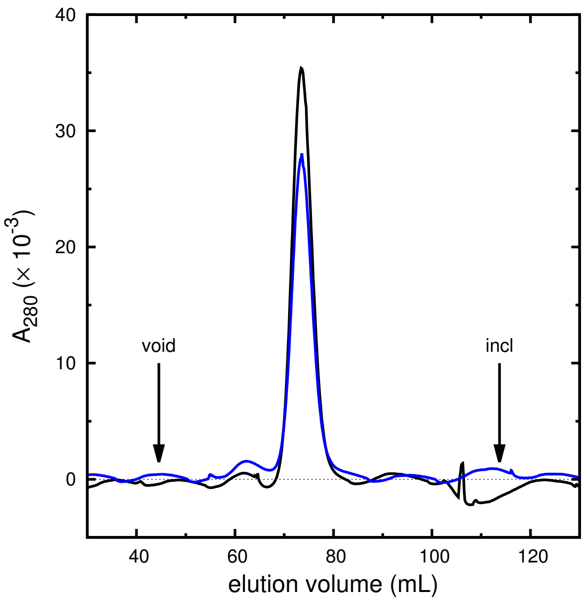

Figure S1. Gel filtration analysis of TpCSH6 (black trace) and TpCSH6-H222Q (blue trace) at 277 K in 50 mM Tris · HCl, pH 8.0, and 100 mM KCl. Molecular size analysis was performed as described previously (Mullins *et al.*, 2013). The arrows indicate the elution volumes for Blue Dextran (void) and acetone (incl) standards. Each elution profile reaches a maximum at 73.6 mL (81 kDa), as expected for a dimer of 43 kDa subunits.

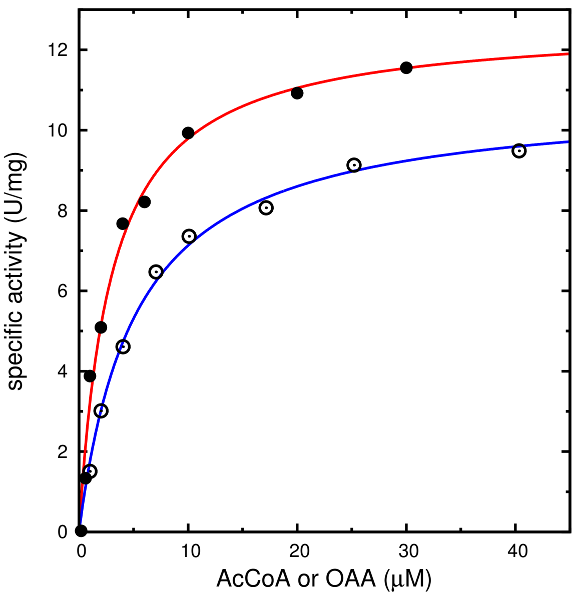

Figure S2. Determination of steady-state kinetic parameters for TpCSH6. Specific activities were measured using the DTNB assay at 298 K for AcCoA at 0.4 mM OAA (filled circles) or OAA at 15 μM AcCoA (open circles). Solid lines depict fits to to the Michaelis-Menten equation: AcCoA, $K_\mathrm{M} = 3.0 \pm 0.4$ μM and $V_\mathrm{max} = 12.7 \pm 0.5$ U/mg; OAA, $K_\mathrm{M} = 5.2 \pm 0.4$ μM and $V_\mathrm{max} = 10.8 \pm 0.2$ U/mg.

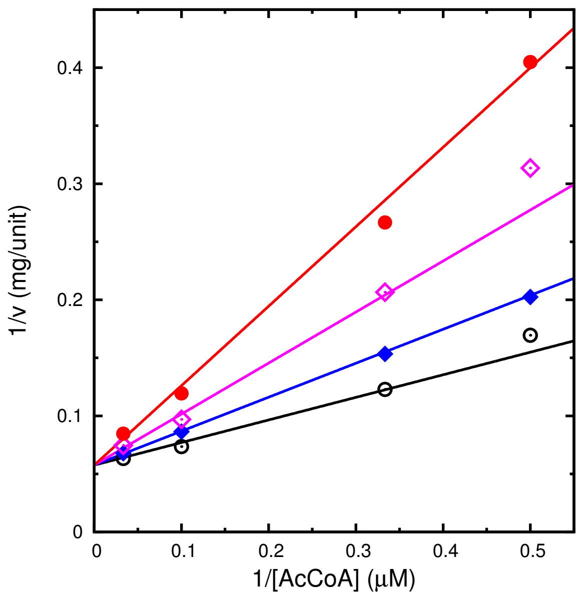

Figure S3. Determination of a competitive inhibition constant (K_i) for AcMX. Global fit parameters were: $K_{\mathrm{M}} = 3.3 \pm 0.2$ μM, $K_i = 19.9 \pm 2.1$ μM, and $V_{\max} = 17.4 \pm 0.3$ U/mg. [Phosphate weakly inhibits TpCS (Kurz *et al.*, 2009).] The solid lines represent curves computed using global fit parameters at 0 (black open circles), 10 μM (blue filled diamonds), 25 μM (purple open diamonds), and 50 μM (red filled circles) AcMX.

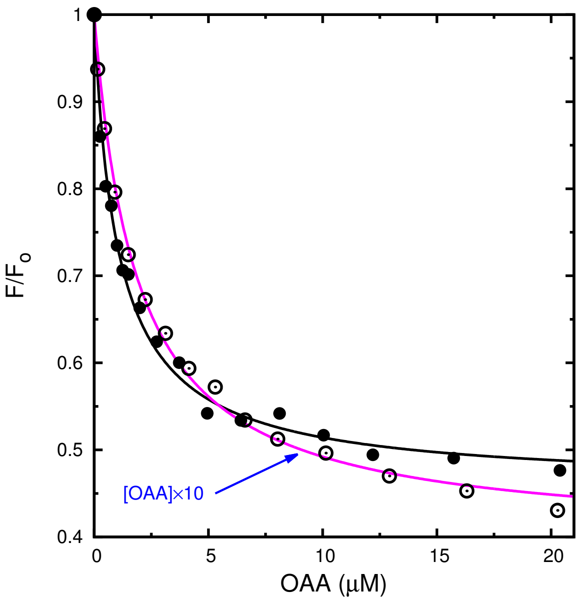

Figure S4. OAA affinities determined by fluorescence titration. OAA titrations were performed by adding aliquots to a constantly stirred 2 mL volume that contained 50 mM EPPS, pH 8.0, 0.1 mM EDTA, and either TpCSH6 or TpCSH6-H222Q. Spectra were recorded 3 min after each ligand addition. The OAA-dependent quenching of TpCSH6 (0.2 μM, filled circles) or TpCSH6-H222Q (1 μM, open circles) were analyzed by fits to Eqn. S2 and Eqn. S1, respectively. Solid lines depict fits for TpCSH6 (black curve) and TpCSH6 (purple curve), divided by F_o: K_d = 0.76 ± 0.06 and 19 ± 1 μM and maximal (e.g., [OAA] $\longrightarrow \infty$) $-\Delta F/F_o$ = 0.541 and 0.603, respectively. Note that [OAA] in the TpCSH6-H222Q titration was 10-fold higher than indicated on the abscissa.

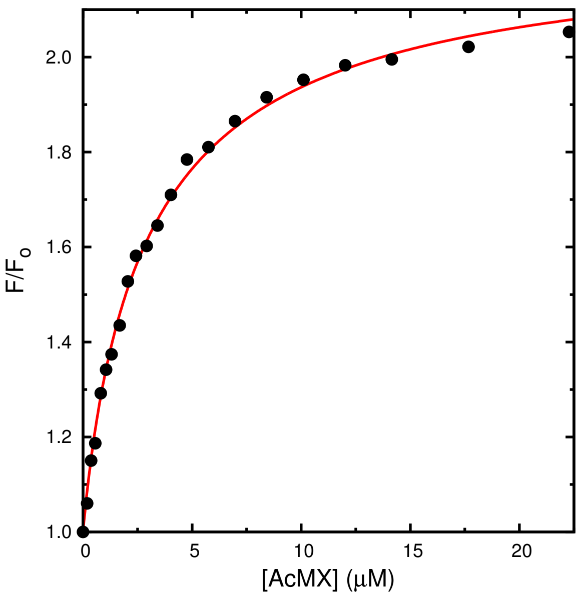

Figure S5. AcMX affinity for TpCSH6·OAA determined by fluorescence titration. Experimental conditions were the same as described in Fig. S4, except that 0.2 mM OAA was also present. The AcMX-dependent increase in F is due to conversion of the fluorescence quencher OAA to CitMX. The solid line depicts a fit of the titration data to Eqn. S2, divided by F_o, with $K_\mathrm{d} = 1.5 \pm 0.1$ μM and maximal $\Delta F/F_o = 1.24 \pm 0.01$.

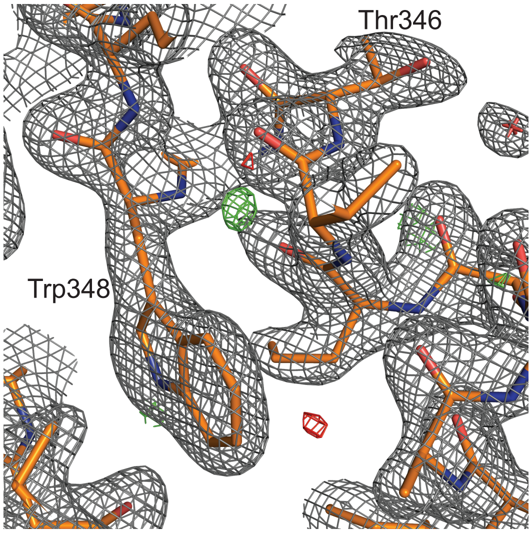

Figure S6. Representative map-model agreement. Electron density maps near Trp348 (subunit C) are shown: σA-weighted 2mFo–DFc (grey mesh, 1.5σ) and mFo–DFc (green and red mesh, $\pm 3\sigma$). The Trp348 rotamer, which is essentially the same as in liganded TpCS structures, is flipped (-171° rotation) relative to the rotamer observed in the structure of TpCS (Russell *et al.*, 1994).

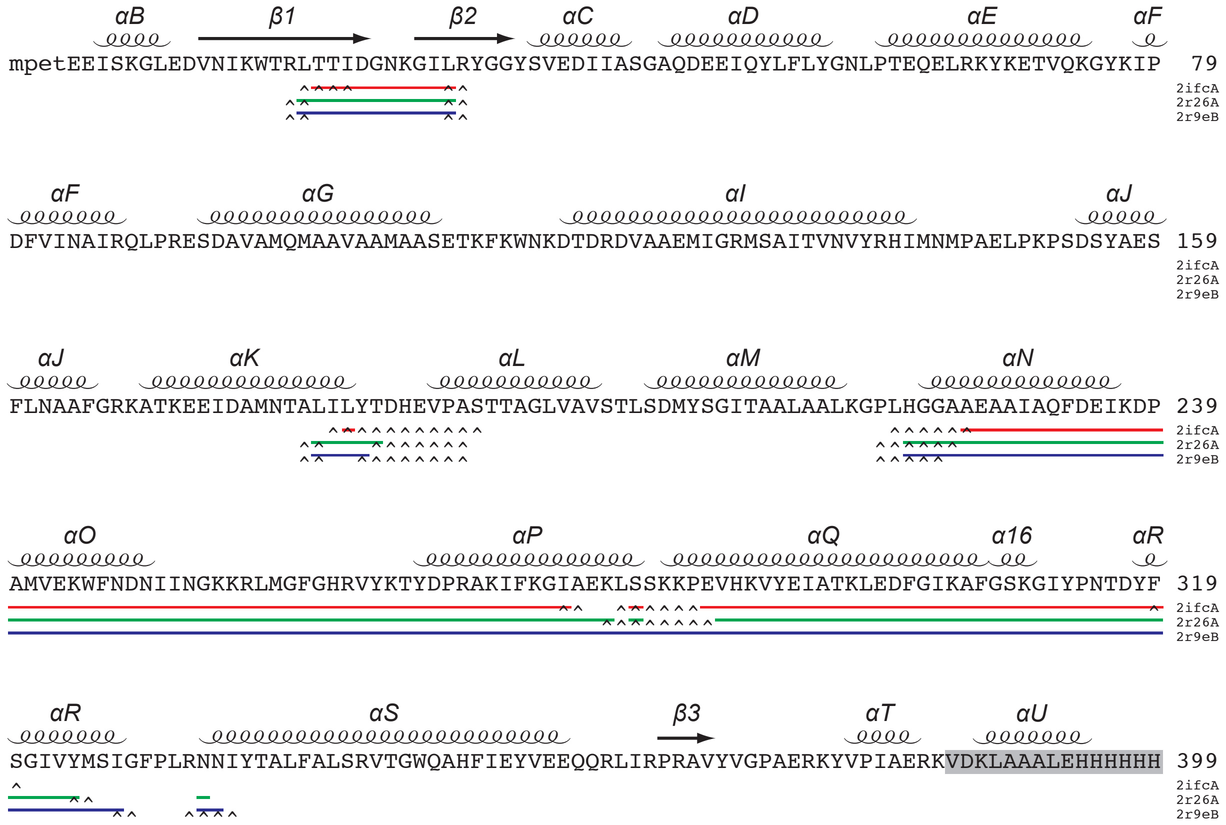

Figure S7. Primary sequence, secondary structure, and domain motions in TpCS(H6). The C-terminal appendage of TpCSH6 is indicated with a grey background. In keeping with prior practice, the initiator codon is assigned the number zero. The elements of secondary structure are given the same designations as in TpCS, apart from a short helix (α16) and C-terminal appendage helix (αU) that were not present in the apo TpCS structure. The colored symbols correspond to mobile domains (underlined) and hinge residues (carets) identified by DynDom comparisons of TpCSH6 with one ligand-bound TpCS subunit.

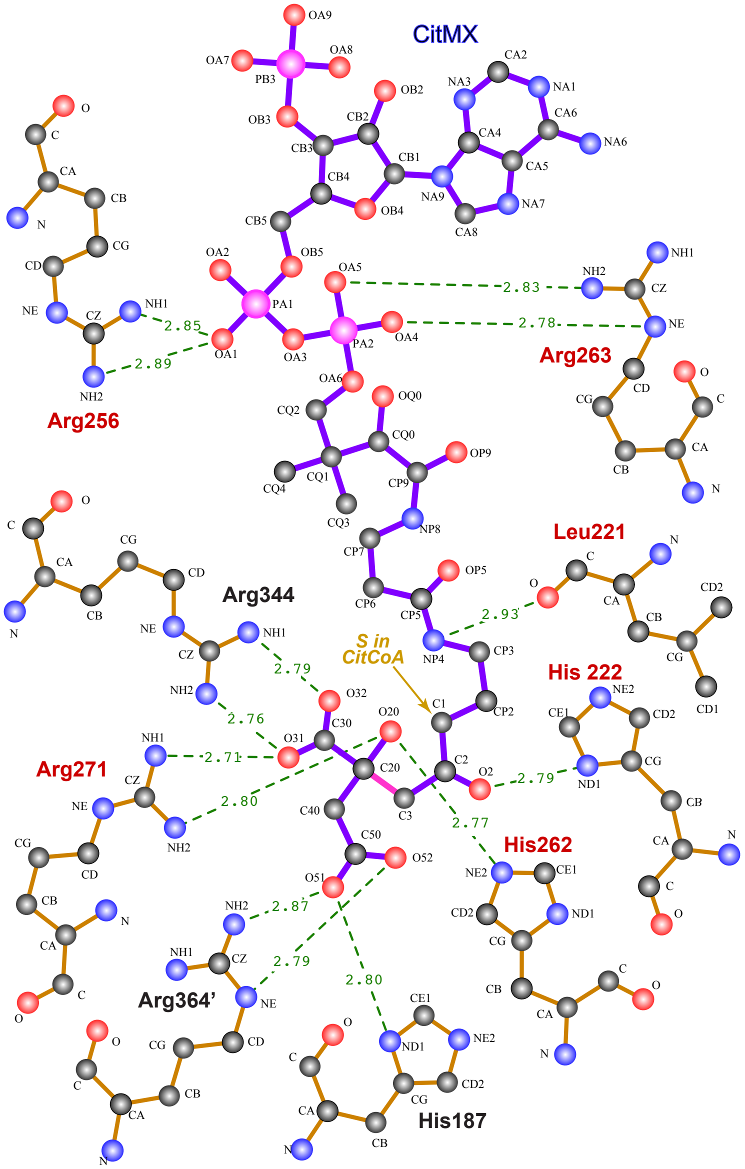

Figure S8. Active site of TpCS · CitMX complex (PDB entry 2r9e) showing selected polar protein-ligand interactions. CitMX differs from CitCoA by an atomic substitution (S ⟶ CH_2) indicated with an arrow. TpCS forms CitMX by condensing AcMX and OAA, forming the bond denoted in magenta. Residues with names shown in red are also involved in binding the C-terminus of TpCSH6 subunit A or D (Fig. 2). The prime denotes a residue from the partner subunit in the TpCS dimer; note that the C-terminus only engages residues from one subunit. Polar contacts with the CoA region by Leu257, Gly259, Phe260, Asn315, and Arg 361 are omitted.
